# DIABRISK - SL Prevention of cardio-metabolic disease with life style modification in young urban Sri Lankan's - study protocol for a randomized controlled trial

**DOI:** 10.1186/1745-6215-12-209

**Published:** 2011-09-26

**Authors:** Mahen Wijesuriya, Martin Gulliford, Laksha Vasantharajah, Giancarlo Viberti, Luigi Gnudi, Janaka Karalliedde

**Affiliations:** 1 Diabetes Association of Sri Lanka, National Diabetes Centre, Colombo, Sri Lanka; 2 Division of Health and Social Care Research, King's College London, UK; 3 Cardiovascular Division King's College London, UK

## Abstract

**Background:**

Urban South-Asian's are predisposed to early onset of type 2 diabetes mellitus (T2DM) and cardiovascular disease (CVD). There is an urgent need for country specific primary prevention strategies to address the growing burden of cardio-metabolic disease in this population. The aim of this clinical trial is to evaluate whether intensive (3-monthly) lifestyle modification advice is superior to a less-intensive (12 monthly; control group) lifestyle modification advice on a primary composite cardio-metabolic end point in 'at risk' urban subjects aged between 5-40 years.

**Methods/Design:**

This is an open randomised controlled parallel group clinical trial performed at a single centre in Colombo, Sri-Lanka. A cluster sampling strategy was used to select a large representative sample of subjects aged between 5-40 years at high risk of T2DM and CVD for the intervention study. We have screened 23,298 (males 47% females 53%) healthy subjects for four risk factors: obesity, elevated waist circumference, family history of diabetes and physical inactivity, using a questionnaire and anthropometry. Those with two or more risk-factors were recruited to the intervention trial. We aim to recruit 4600 subjects for the intervention trial. The primary composite cardio-metabolic end point is; new onset T2DM, impaired glucose tolerance, impaired fasting glycaemia, new onset hypertension and albuminuria, following 5 years of intervention. The effect of the intervention on pre-specified secondary endpoints will also be evaluated. The study will be conducted according to good clinical and ethical practice, data analysis and reporting guidelines.

**Discussion:**

DIABRISK-SL is a large population based trial to evaluate the prevalence of diabetes, pre-diabetes and cardio-metabolic risk factors among young urban Sri-Lankans and the effect of a primary prevention strategy on cardio-metabolic disease end points. This work will enable country specific and regional cardio-metabolic risk scores to be derived. Further if the proposed intervention is successful the results of this study can be translated and implemented as a low-cost primary prevention tool in Sri-Lanka and other low/middle income developing countries.

**Trial registration:**

The trial is registered with the World Health Organisation and Sri-Lanka clinical trial registry number SLCTR/2008/003

## Background

Type 2 Diabetes Mellitus (T2DM) and associated cardiovascular complications pose a major health-care burden worldwide and present a significant challenge to patients, health-care systems, and national economies [1]. Nearly two thirds of the estimated 285 million people with diabetes (predominantly T2DM) live in low and middle income countries [1,2]. Recent data indicates that South Asia is one of the major sites of this epidemic of T2DM with a projected 72% increase in the number of subjects with T2DM in the next 20 years [1]. In parallel there is an epidemic of pre-diabetes [impaired glucose tolerance (IGT) and impaired fasting glycaemia (IFG)], with prevalence rates between 10-15% reported in South Asian adult populations [1].

T2DM and IGT are associated with a significantly increased risk of cardiovascular disease (CVD) with South Asian's particularly predisposed to early onset of T2DM and CVD [1]. In South Asia almost a third of T2DM cases will be in those aged below 45 years [1]. Both CVD and T2DM may share a common pathogenesis and indeed retain many common risk factors/features [1].

Sri Lanka is a middle income country in Asia with a population of 20 million. Nearly 40% of Sri Lankans are aged below 40 years and 25% are aged below 18 years [3]. There has been a rapid urbanisation in recent decades with an estimated 30% of the population now living in urban areas [3]. The most recent national study in subjects aged over 20 years indicated a population prevalence of dysglycaemia (defined as T2DM or IGT or IFG) of 20% which rose to 30% in urban areas [4]. Physical inactivity, raised body mass index (BMI) and central obesity along with urban living were strongly associated with the increased risk of dysglycaemia.

The majority of lifestyle modification (LSM) intervention studies/programmes on preventing T2DM have been done in older pre-diabetic subjects with mean ages between 45-50 years [5-7]. A recent meta-analysis suggested that in subjects with IGT, the number needed to treat to prevent one case of T2DM was 10.8 with anti-diabetic drugs as compared to 6.4 with LSM [8]. Furthermore long term 'legacy' benefits of LSM which can last up to 14 years following the cessation of LSM have been reported [9,10].

To date there is limited information on the prevalence of T2DM and cardio-metabolic risk factors in young urban populations from Sri-Lanka or other similar South-Asian countries. Moreover the effect of a LSM programme on cardio-metabolic disease end-points has not been hitherto evaluated in this unique population.

### General objective

The objective of the trial is to test the hypotheses that intensive (3 monthly) LSM advice will be superior to less intensive (12 monthly) LSM advice in preventing cardio-metabolic disease in 'at risk' young urban Sri Lankan subjects.

### Primary objective/end-point

The primary objective of the trial is to determine whether intensive (3 monthly) LSM advice will be superior to less intensive (12 monthly) LSM advice in reducing the primary composite cardio-metabolic end point of new onset T2DM, impaired glucose tolerance, impaired fasting glycaemia, new onset hypertension and albuminuria at 5 years.

### Secondary objective/endpoints

Secondary end-points include; the individuals components of the primary composite end-point, incidence of metabolic syndrome as defined by the International Diabetes Federation (IDF) [11,12], incidence of pre-hypertension as defined by Joint National Committee on Prevention, Detection, Evaluation, and the Treatment of High Blood Pressure (JNC-7) [13], regression of IGT and IFG, [IGT and IFG will be defined as per World Health Organisation (WHO) and American Diabetes Association criteria [14,15]] and changes in: weight, BMI, waist circumference (WC), fasting triglycerides, fasting high density lipoprotein (HDL) and low density lipoprotein (LDL) cholesterol, serum creatinine and estimated glomerular filtration rate (GFR), physical activity as measured by the International physical activity questionnaire (IPAQ)[16], depression, as measured by PHQ-9 questionnaire, [17], and psychological stress, as measured by perceived stress scale [18]. Adherence to diet and physical activity will be self-reported based on weekly pattern and evaluated at study visits. The effects of baseline risk factors on the development of end-points will also be evaluated in analyses.

Quality of life measures will be evaluated using a validated short version of the multidimensional WHO Quality of Life questionnaire (WHOQOL-Bref) [19]. Health economic analyses by the Public Health Department University of Colombo with advice and guidance from Division of Social Care and Policy at King's College London is an area we plan to develop utilising models described in recent studies [20].

## Methods/Design

### Study design

**Figure 1:**
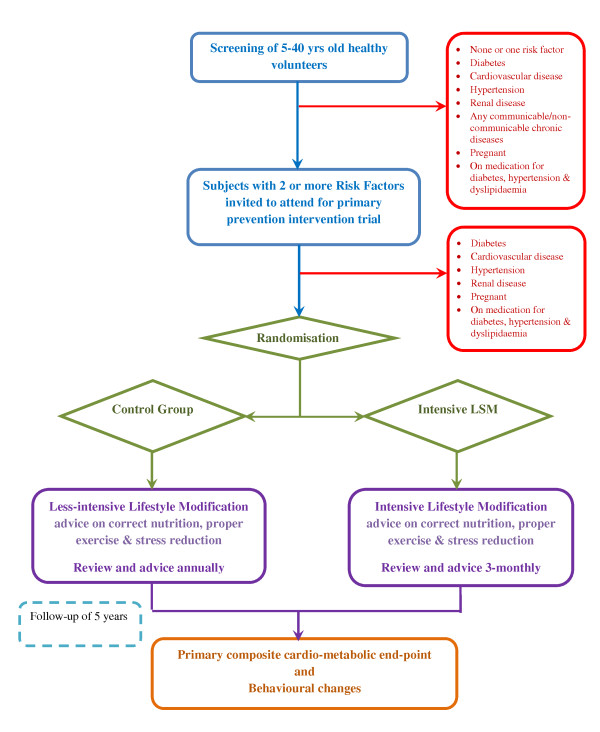
**Trial flow chart**. Figure 1 describes trial flow chart from screening to randomisation and end point evaluation at 5 years.

DIABRISK-SL is an open randomised controlled parallel group clinical trial evaluating the effect of intensive (3-monthly; study group) *vs*. less-intensive (12-monthly; control group) LSM advice on a primary composite cardio-metabolic end-point (new onset T2DM, IGT, hypertension, and albuminuria) in 'at-risk' young urban subjects aged between 5-40 years. The two arms of the study have 1:1 allocation ratio and follow up is over 5 years. Figure 1 details the trial flow chart.

### Ethics and Trial Registration

The study is funded by IDF BRIDGES, an IDF project supported by an educational grant from Eli Lilly Diabetes and a grant award from the Diabetes Association of Sri Lanka (DASL). The study protocol is approved by Ethical Review Committee of Sri Lanka Medical Association (ERC 07-010). The trial is registered at Sri Lanka Clinical Trials Registry (Clinical Trial Registration Number SLCTR/2008/003), available online at: http://apps.who.int/trialsearch/Trial.aspx?TrialID=SLCTR/2008/003.

### Study population and recruitment

We screened a population representative of the general population of Colombo District, the most populous urban district in Sri-Lanka, aged between 5 to 40 years to identify the prevalence of four risk-factors namely first degree family history (FH) of T2DM, physical inactivity, raised BMI and raised WC. The sampling strategy was designed to provide a sample that was representative of the age and sex distribution of the general population aged between 5-40 years in the Colombo district [3]. A list of schools, workplaces, universities and community organisations was made that were representative of the organizations and population aged between 5 to 40 years obtained from the most recent national census results. This list comprised of 65 organisations and each of these organizations was individually approached and eligible participants were invited to take part in the screening study. The screening survey took place between 1^st ^January 2008 and 30^th ^June 2009. The age and sex distribution of the screened population was regularly compared with the distribution of the reference population to ensure that the screened population was representative of the distribution of the general population aged between 5 and 40 years in the Colombo district. More than 95% of subjects and institutions approached agreed to participate. Subjects with a known history of T2DM, CVD, hypertension or diabetes and pregnant women were excluded from the initial sample. Subjects on lipid lowering drugs or anti-diabetic or anti-hypertensive drugs were also excluded.

Subjects with any active communicable or non-communicable chronic disease (NCD) such as cancer, asthma or other forms of chronic lung disease, depression, and tuberculosis were also excluded. A screening questionnaire (see additional files 1) was used to determine first degree family history of T2DM and the physical activity of the individual participants. Further information regarding socio-demographic factors was also collected.

The screening study aimed to identify four risk-factors: physical inactivity (<30 minutes continuous exercise for <3 days/week), raised WC (central obesity) defined as WC in subjects between 5-17 yrs ≥91^th ^percentile, 18-40 yrs: females ≥80 cm and males ≥90 cm, first degree FH of T2DM and raised BMI defined in subjects aged 5-18 yrs of a BMI value greater than internationally standardized age and sex specific percentile cutoffs and between 18-40 yrs as BMI ≥23 kg/m^2 ^[21,22]. These four factors were chosen on the basis of a pilot study that identified these risk-factors as being highly prevalent in 1239 subjects aged below 40 years with a new diagnosis of T2DM who attended the National Diabetes Centre in Colombo between 2005-2007 and are supported by recent data from comparable South Asian and Sri Lankan populations [1,4,22,23].

### Inclusion Criteria for primary prevention intervention trial

Subjects aged 5-40 years with two or more risk factors for cardio-metabolic disease. The risk factors for selection included two or more of the following;

• ***First degree FH of T2DM***

• ***Physical inactivity***

• ***Raised BMI***

• ***Raised WC***

### Exclusion Criteria

#### During screening

• Subjects with no or one identifiable risk factor

• Subjects with diagnosed end-points (T2DM, CVD, hypertension and renal disease)

• Subjects on any form of medication used for the treatment of diabetes, hypertension, renal disease or dyslipidaemia

• Any active communicable or NCD such as cancer, asthma or other forms of chronic lung disease, depression, tuberculosis.

• Pregnant women

#### During the primary prevention intervention trial

• Subjects who reach one or more of the following cardio-metabolic end-points; T2DM, CVD, hypertension, and albuminuria.

• Subjects initiated on any form of medication used for the treatment of diabetes, hypertension, renal disease or dyslipidaemia by their family physician or other medical physician.

• Pregnancy at any stage of the study

### Randomisation

Subjects who satisfy the selection criteria will be randomised into two intervention group using a computer generated randomisation schedule/sequence (SPSS 10.0) prepared by an independent party (Department of Statistics and Public Health Colombo University). Randomisation was performed sequentially using a SPSS programme function which enables this process and is supervised by an independent statistician from the Department of Statistics and Public Health at Colombo University, Sri Lanka.

#### Procedures

The following procedures and measurements will be performed at baseline prior to randomisation to one of the two groups.

Height is measured using a portable stadiometer (Seca France) and taken to the closest 0.1 cm, and weight is measured without footwear in light clothing to the nearest 100 g with an electronic weighing scale (Seca France). Both instruments are calibrated weekly. BMI is calculated as weight in kilograms divided by height squared in meters. WC is measured using a graduated measuring tape (Seca France), calibrated weekly, with a locking device to the nearest 0.1 cm. WC is taken at the mid-point between the iliac crest and the last rib in expiration. Blood pressure is measured by automatic oscillometry from the non-dominant arm after 5 minutes rest with the mean of two measurements recorded (Riester Ri-Champion Riester, Jungingen, Germany). WHO approved growth charts for male and female children will be used [24].

Fasting plasma glucose and a standard 2-hour 75 g oral glucose tolerance test (OGTT) is performed in adults and glucose solution 1.75 g/kg body weight, to a maximal dose of 75 g is given to those below 16 years following a 12-hour fast as per WHO guidelines [14]. Plasma Glucose, total cholesterol, high density density lipoprotein (HDL), low density lipoprotein (LDL), triglycerides and serum creatinine will be measured by enzymatic colorimetry (Vitros MS 250, Ortho Clinical Diagnostics, Johnson and Johnson, Co, Rochester USA). Fasting plasma insulin will be measured using radioimmunoassay (Immulite, Siemens, Surrey, UK), which has a sensitivity of 4 μU/ml (<24 pmol/l) and intra and interassay coefficients of variation <8%. Insulin resistance will be calculated using the homeostasis model assessment of insulin resistance (HOMA-IR) [25]. Urine albumin to creatinine ratio will be measured by immunoturbidemtry (Vitros 5,1 FS Chemistry System, Ortho-Clinical Diagnostics, Johnson and Johnson Company, Hampshire, United Kingdom) from an early morning urine sample. In subjects below 18 years the Schwartz equation will be used to estimate glomerular filtration rate (GFR) from serum creatinine values [26] and in subjects above 18 years the Modification of Diet in Renal Disease (MDRD) Study equation will be used to estimate GFR [27].

### Intervention and goals

Intensive LSM group: The main goals of the intensive group delivered three monthly on an individual basis are:

Dietary advice and support: individualised advice is given on the need to have a well balanced food intake and to maintain appropriate body weight in those with normal BMI and WC. In subjects with a raised (age and gender appropriate) BMI and WC a goal of >5% weight loss is set concordant to targets in recent studies [5-8]. Dietary advice on avoiding a high intake of food containing simple sugars, refined carbohydrates and high saturated fats (total fat intake <20 g/day) and advice to increase intake of healthy natural foods which are fibre-rich-whole grains, legumes, vegetables and fruits is given. A list of refined foods to be avoided is provided. Subjects are advised on the importance of regular meals and to avoid delaying or missing meals.

Exercise; Subjects with physical inactivity at baseline are advised to increase physical activity to at least 30 minutes of continuous exercise a day for more than 5 days of the week. Individualised exercise advice is given depending on the needs and facilities available to the subject. Children are advised on need to play at least for 1 hour every day. All subjects are advised to minimise sedentary activity such as watching television. For children, the LSM advice will be given to the parent and the child jointly. To ensure that the intervention does not hamper normal growth, assessment of the child's growth (height and weight) will be performed at each visit and these measures compared to the previous values on the child's growth chart; and if there is any medical and or parental concerns regarding growth, the child will be referred to a paediatrician for further evaluation and assessment.

The dietary and exercise goals are comparable to those set in the Indian Diabetes Prevention Programme (IDPP) [6]. All subjects have a three monthly telephone contact to assess progress and reiterate goals. The total number of one to one contacts is 4 per year. The basis of LSM advice is made on the transtheoretical model of behaviour change [28] which postulates that individuals are at different stages of readiness to adopt a new behaviour and that individuals are required to progress through various 'stages of change'. Evidence suggests that providing individualised interventions tailored to a subject's stage of behaviour change i.e. stage matched, as done in this study, are more effective than providing the same intervention to all subjects [29].

Control group: Less intensive (annual) lifestyle advice: Identical dietary and exercise advice as outlined above are given to all subjects in the control group; however this is done on a 12 monthly basis. The goals and targets set for diet and exercise are identical to those in the intensive group but these are evaluated and reinforced annually in one individualised face to face contact session with an educator.

### Statistical methods and power calculation

Approximately 2300 subjects with ≥2 risk-factors are required in each group (4600 subjects in total) to detect a 25% relative risk reduction at 5 years on the primary composite cardio-metabolic end-point of new onset T2DM, IGT, IFG, hypertension, albuminuria, renal and CVD events with intensive LSM. The study is powered at the 90% level with a type-1 error of 0.05 (2 sided) assuming a 15% incidence of the primary end-point in the control group and including allowance for 25% drop-out of subjects. The proposed 25% relative risk reduction of the composite outcome is derived from the effects of intensive LSM programme reducing the relative risk of new onset T2DM in South Asian IGT patients [1,6].

There will be a pre-specified interim analysis of the composite primary and secondary endpoints at 2 years, as requested by one of the funding bodies (IDF BRIDGES). This analysis will be performed by an independent statistician and the trial investigators will remain blinded to the full data analyses and interim results. All data related to interim analysis will directly forwarded to funding body by the independent statistician and will be strictly confidential and not for public dissemination/publication.

Descriptive statistics will be used for the analysis of demographic and clinical features of the cohort. Two-sided t-tests and chi-square (χ^2^) tests will be used to analyze the comparison between the groups at baseline and during follow-up. Survival curves will be calculated to estimate the cumulative incidence of the composite primary end-point and secondary end-points. The difference between the groups in the primary end-point will be tested by means of the two-sided log-rank test. All analyses of end-points are based on the intention-to-treat principle. Analyses will be performed using STATA software version 10 (Stata Corp, College Station, TX, USA).

### Definition of primary cardio-metabolic end-points

▪ **New onset T2DM **as indicated by either a fasting plasma glucose of ≥7.0 mmol/l and/or a 2-hr plasma glucose concentration of ≥11.1 mmol/l during an annual follow-up confirmed by repeat 75 g OGTT as per World Health Organization (WHO) criteria [14] or initiation anti-diabetic treatment by the subject's family physician following confirmatory tests.

▪ **New onset IGT **is defined as a plasma fasting glucose <7.0 mmol/l and 2-h glucose 7.8-11.0 mmol/l and IFG as fasting plasma glucose between 6.1 to.9 mmol/l or 5.6 to 6.9 mmol/l [14,15].

▪ **New onset hypertension **is defined as mean brachial blood pressure from 2 readings taken following 5 minutes rest ≥140/90 mmHg or initiation of anti-hypertensive treatment commenced by the subject's family physician as per JNC-7 criteria [13]. In those below 18 years hypertension is defined as blood pressure that is, on repeated measurement, at the 95^th ^percentile or greater adjusted for age, height, and sex [13].

▪ **New onset albuminuria is defined as **urine albumin/creatinine ratio ≥2.5 mg/mmol in males and ≥3.5 mg/mmol in females from early morning sample confirmed on one repeat test.

▪ **New onset renal impairment is **defined as estimated GFR <60 ml/min or doubling of serum creatinine from baseline.

▪ **CVD events **are defined as fatal and not fatal events of Myocardial infarction (MI): Congestive heart failure (CHF): death due to clinical, radiological or post-mortem evidence of CHF, Stroke, vascular revascularisitation and admission for unstable angina.

#### Independent advisory and data monitoring group

An independent external expert advisory and monitoring board has been formulated which includes two experts from the MV-Diabetes Research Centre and Hospital in Chennai India (IDPP trial site) and one Sri Lankan expert from the Department of Community medicine, University of Colombo. This board will regularly review the progress of the DIABRISK-SL study and will also independently adjudicate on CVD events.

## Discussion

This study will be the first large population based study to determine the effect of a primary prevention LSM strategy on cardio-metabolic risk in a young urban population. This work will enable the characterisation of the natural history of cardio-metabolic disease in a unique at-risk population hitherto not studied in such detail or number. Importantly research project such as DIABRISK-SL enhance awareness of diabetes and associated risks amongst the study subjects, their friends and families. Such public health promotion is invaluable in countries with limited financial resources. The proposed intervention programme if successful may be implemented as a low-cost primary prevention tool in Sri Lanka and other similar low and middle income countries.

Our study will also determine the prevalence of T2DM, pre-diabetes, and cardio-metabolic risk factors in young urban Sri Lankans. We have also aimed to develop a method for identifying a sample at increased risk of T2DM and CVD for a randomised controlled trial. We hope to show that a simple screening strategy based on self-reported physical inactivity, family history of T2DM in first degree relatives, as well as obesity and increased WC provides an efficient method for identifying a sample with a high frequency of cardio-metabolic risk. More recently the entity of metabolic syndrome as a disease syndrome has been questioned [30]. However it is irrefutable that individual risk factors for CVD and T2DM cluster and inter-relate [11,12]. Furthermore in its purest form, as we will report with the exclusion of those with known T2DM or CVD and with detailed description of the individual risk components, it is a pre-morbid condition that could be useful in developing country specific public health intervention strategies [30].

This study will have the largest urban cohort below the age of 40 years in South Asia evaluated for cardio-metabolic risk. Our results will raise awareness of growing burden of T2DM and obesity in developing countries that is of major public health and economic importance and which requires urgent remedial actions [1,31]. The results of DIABRISK-SL will help advise and assist in the formulation of a national policy and guidance document for combating T2DM and associated non-communicable diseases in Sri Lanka [32].

## Competing interests

The authors declare that they have no competing interests.

## Authors' contributions

MW is the principle investigator (PI) for the DIABRISK-SL study and he is the trial coordinator in Sri Lanka. GV is an advisor to the PI and other study investigators. MW, GV, LG and JK were actively involved in the clinical trial management, intervention design and methodology for this project. MG will perform all statistical analyses and provide support with data interpretation and research methodology. LV is responsible for the overall day to day management of the trial and involved in the education and training of the lay educators for this study. MW, LG, JK and LV were involved in drafting the study protocol. All authors critically evaluated the article for content and approved the final version.

## Supplementary Material

Additional file 1 **Screening questionnaire**. Screening questionnaire used to determine first degree family history of T2DM and the physical activity of the individual participants. Click here for file
