# Supplementary material for: DIABRISK - SL Prevention of cardio-metabolic disease with life style modification in young urban Sri Lankan's - study protocol for a randomized controlled trial
Source: Trials. 2011 Sep 26;12:209. doi: 10.1186/1745-6215-12-209 (PMC3197497; doi:10.1186/1745-6215-12-209)
Supplement: Additional file 1 — Screening questionnaire. Screening questionnaire used to determine first degree family history of T2DM and the physical activity of the individual participants. [file 1745-6215-12-209-S1.PDF]

Stage 1: Questionnaire

Code No (initial) [ ] Date [ ]

----

1. Name: ……

2. Address (permanent) Address (temporary**)**

……………………………………………………… ……………………………………………….

……………………………………………………… ……………………………………………….

3. Province. ……………………………………………… ……………………………………………….

4.Tel number/s: ……………………….. **Mobile no:** ………………………………

5. Date of Birth:

D	D	M	M	Y	Y

6. Sex:

Male	
Female	

7. Current health status (Mark with X):

Do you currently have	Yes	No	*Do not know*
a. Elevated Blood sugar/Diabetes			
b. Heart disease(Heart attack/MI, Angina, Bypass surgery, angioplasty)			
c. High blood pressure (Hypertension)			
d. High cholesterol(Dyslipidaemia)			
e. Kidney disease (Diabetic/Hypertensive)			
f. Pregnancy			
*g. Any other long term illness**			

Please specify the details of *:
……..

8. Family history of Diabetes (Mark with X)

[ ] Mother [ ] Father [ ] Brother/Sister

[ ] Son/Daughter [ ] Paternal/Maternal Aunt/uncle [ ] Paternal/Maternal Grand Parent

9. Physical activity (Mark with X)

	Yes	*No*
Exercises : 03 times a week , minimum of 30 minutes continuous (PT/Gym)		
Active Sport: 03 times a week , minimum of 30 minutes continuous		
Walking, Cycling: 03 times a week , minimum of 30 minutes continuous		
Any other physical activity(specify)		
None		

10. Official use only

Height (cm) [ ] Waist (cm) [ ] Weight (kg) [ ]

Letter to the Participant/ Parent /Guardian

Dear Participant/ Parent /Guardian
Evaluation of Risk Factors in the Development of Type 2 Diabetes and Cardiovascular Disease in a Young Urban Population in Sri Lanka.

Diabetes and heart disease are rapidly increasing in prevalence in our country. Both these illness arise in early life and often remain dormant till adult life.

Diabetes Association of Sri Lanka in collaboration with King's College, London, UK are conducting a survey of young persons in Sri Lanka between the age of 10-40 years to determine the presence of risk factors in the development of the above conditions. This involves a completion of a simple questionnaire attached overleaf.

We would sincerely request you to give your consent for participation in this valuable and unique study which will benefit you/ your child as it is intended to be used for prevention of the conditions mentioned above through early identification and advice on life style modification.

Please be good enough to sign and return the consent form and the questionnaire.

Thank you,
Yours faithfully,
Principal Investigators.

Dr Mahen Wijesuriya MD (Cey) FRCP(Lond) FCCP(Cey)
Hon. Director National Diabetes Centre
Dr Thilinie Jayasekera
Senior Medical Officer – Research,National Diabetes Centre

Attachment 2
Stage 1: Written Consent form to be completed by Participant /Parent /Guardian

I………..

Participant/parent/guardian of

Ms/Master..

here by declare /certify that the researchers from the National Diabetes Centre have explained in detail the proposed procedure of the study "**Evaluation of Risk Factors in the Development of Type 2 Diabetes Mellitus and Cardiovascular Disease in a Young Urban Population in Sri Lanka"** and I give my consent for my daughter/son to take part in same.

………………………………. …………………….

Signature of the Participant/ parent/Guardian Date
